# Solitary fibrous tumor in the subcutaneous soft tissues of the left abdominal wall: A case report

**DOI:** 10.1002/ccr3.2217

**Published:** 2019-06-04

**Authors:** Shuanhu Wang, Jiajia Guan, Mulin Liu, Shiqing Li, Congqiao Jiang

**Affiliations:** ^1^ Department of Gastrointestinal Surgery The First Affiliated Hospital of Bengbu Medical College Bengbu China

**Keywords:** abdominal wall, solitary fibrous tumor, spindle cell sarcoma

## Abstract

Solitary fibrous tumor (SFT) derived from the abdominal wall is rare. We report a case of SFT in the abdominal wall. When abdominal computed tomography reveals soft‐tissue mass and analysis of preoperative needle biopsy reveals spindle cell tumor, the possibility of SFT should be considered.

## 1 BACKGROUND

Solitary fibrous tumor (SFT) is a rare spindle cell neoplasm believed to be the source of pleural mesothelial tissue. Klemperer and Rabin^1^ first described the tumor in 1931, and it has been reported in various extrapleural locations, such as intracranial and spinal cord,^2^, ^3^ and face.^4^ To our knowledge, SFT in the subcutaneous soft tissues of the abdominal wall has not been reported.

## 2 CASE PRESENTATION

**Figure 1:**
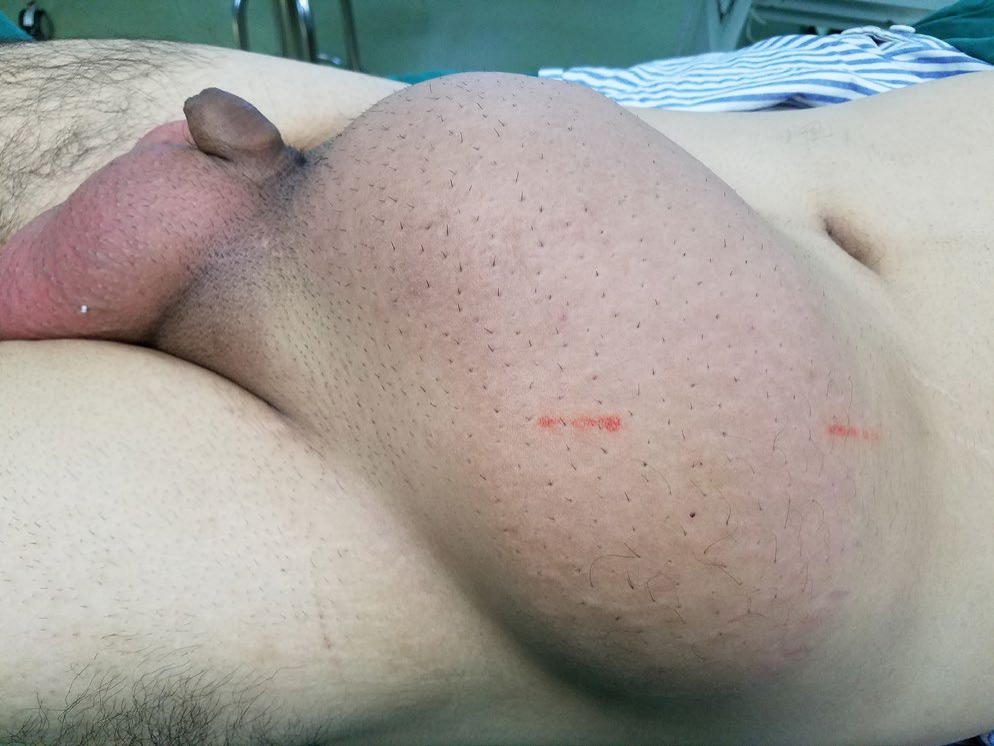
Huge soft‐tissue mass in the left abdominal wall

**Figure 2:**
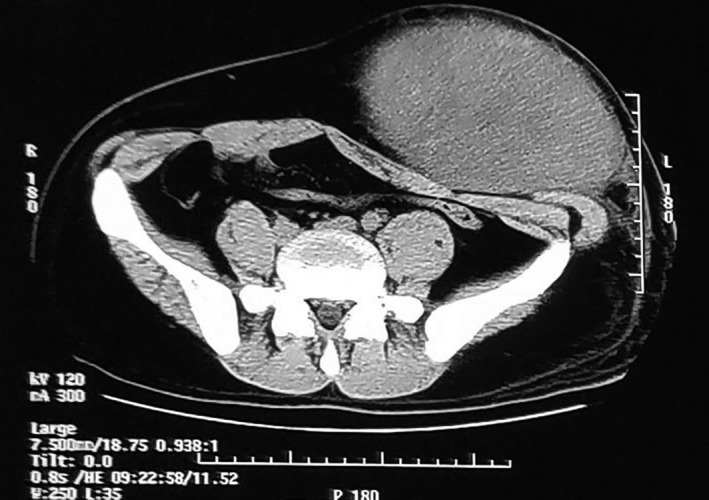
Preoperative lower abdominal computed tomography revealing soft‐tissue mass in the left abdominal wall

**Figure 3:**
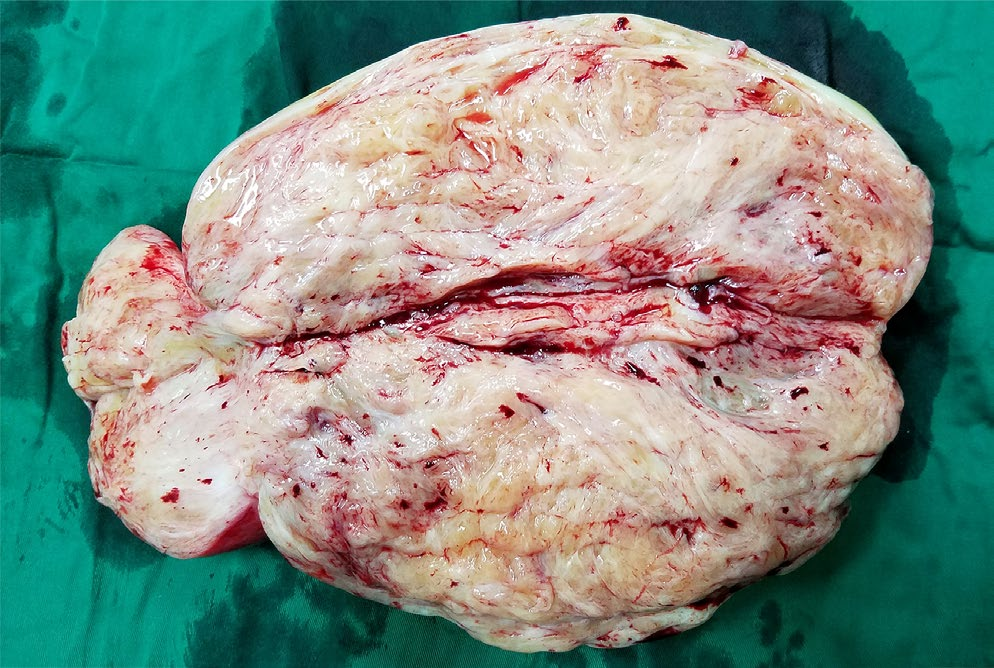
Resected mass

**Figure 4:**
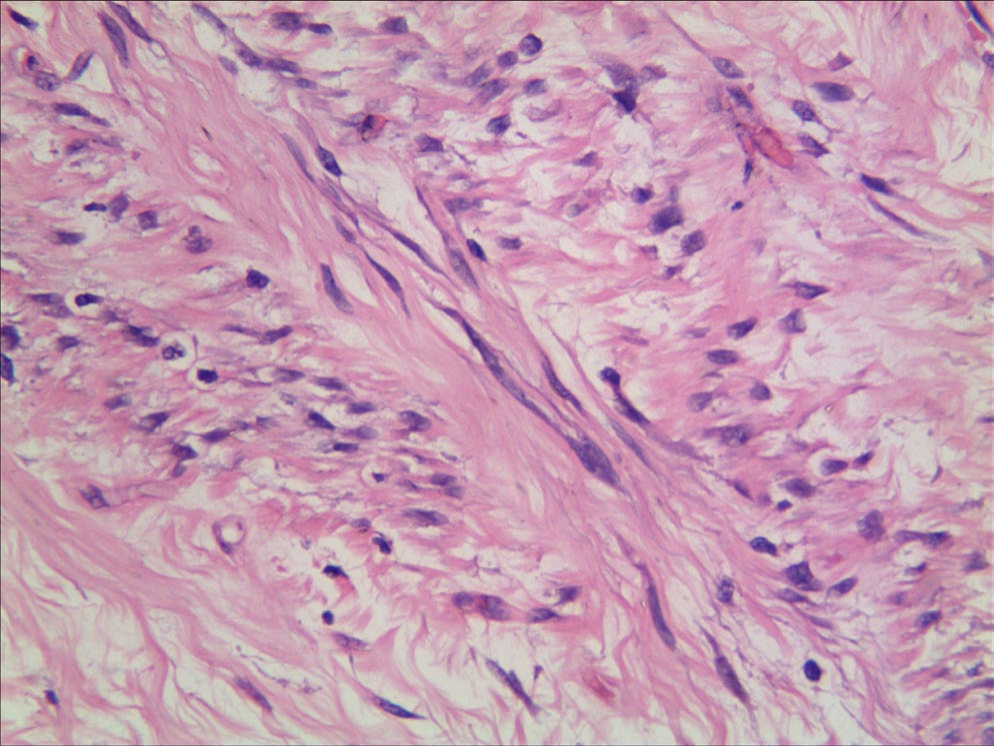
Histological section of resected mass revealing dense and sparse areas of spindle cells and collagen bundles in the stroma (hematoxylin‐eosin ×400)

A 26‐year‐old man presented with a rapidly growing mass in the left abdominal wall (Figure 1). The mass was well demarcated and mobile, and had slight changes in skin color. It was first noticeably present one year earlier, when it was 2 cm in diameter. It had grown rapidly over the next eight months. Abdominal computed tomography demonstrated a solid mass measuring 13 × 27 cm. It looked like oval bump with uniformity signal, laying in the subcutaneous soft tissues, and the CT value was 40 HU (Figure 2). Fine‐needle aspiration revealed spindle cell tumor. At operation, the mass was completely encapsulated in subcutaneous tissue. It was easily separated from the adjacent tissue and was completely removed. Gross pathology examination revealed a white‐brown‐yellow mass (Figure 3). Microscopic examination revealed dense and sparse areas of spindle cells, collagen bundles in the stroma, and thin‐walled branching vessels (Figure 4).

**Figure 5:**
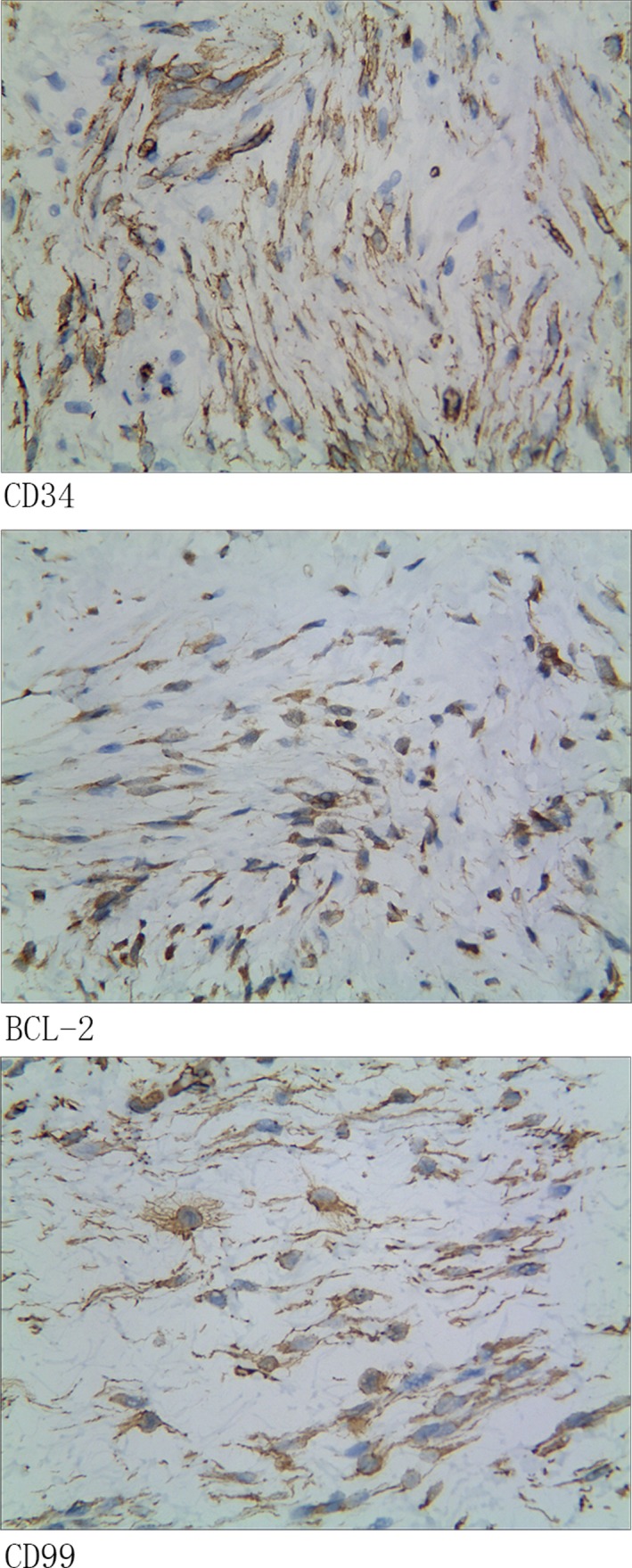
Immunohistochemistry revealing the tumor stained for CD34, BCL‐2, and CD 99 antigens (×400)

In immunohistochemical staining, the tumor was positive for CD34, B‐cell lymphoma 2 (BCL‐2), and CD99 antigens (Figure 5). Stains for smooth muscle actin and S‐100 were negative. The Ki‐67 proliferation index was 3%. Based on the histological and immunohistochemical findings, a diagnosis of SFT was made. The patient had no postoperative complications, and there was no evidence of recurrence at 12‐month follow‐up.

## 3 DISCUSSION

Solitary fibrous tumor is a rare disease, mostly described in case reports. It is difficult to diagnose and often misdiagnosed as other kinds of spindle cell tumors, especially synovial sarcoma.^5^ Immunohistochemical markers, such as CD34, CD99, BCL‐2, smooth muscle actin, S100 protein, vimentin, and CD117, are commonly used to characterize SFT. The SFT immunophenotype (CD34, BCL‐2, and/or CD99 expression) by itself is not specific and may be lost during disease progression.^6^ Some studies have claimed NGFI‐A‐binding protein 2 (NAB2)‐signal transducer and activator of transcription 6 (STAT6) fusion gene, which can be readily detected by STAT6 immunohistochemistry,^7^, ^8^, ^9^ to be hallmarks of SFT. Combined immunohistochemical positivity for STAT6, CD34, CD 99, and Bcl‐2 is supportive of the diagnosis of SFT.^10^

Most often, SFT is a slow‐growing mass with average diameter of about 10 cm.^11^ However, our patient's tumor grew rapidly, to nearly 30 cm, with about a 15‐fold volume increase in 8 months, without injury, or other stimulating factors to explain the increase. The patient hardly ever left home, but we cannot find an association between tumor enlargement and inactivity.

Preoperative needle biopsy was of limited value in our patient as it did not distinguish between SFT and other spindle cell tumors. Complete surgical resection is the main treatment for SFT^12^; radiation therapy or adjuvant chemotherapy has not been beneficial,^13^ and our patient did not receive these treatments. SFT greater than 10 cm is more likely to metastasize than are smaller tumors.^14^ Our patient has no evidence of recurrence after 12‐month follow‐up, but recurrence of SFT has been reported as long as 14 years after resection, so very long‐term follow‐up may be necessary.^15^, ^16^

## 4 CONCLUSIONS

When abdominal computed tomography reveals soft‐tissue mass in the abdominal wall and analysis of preoperative needle biopsy reveals spindle cell tumor, the possibility of SFT should be considered.

## CONFLICT OF INTEREST

None declared.

## AUTHOR CONTRIBUTION

Wang S: performed the operations, reviewed the literature, and drafted the manuscript. Guan J: collected the data and helped to draft the manuscript. Liu M: performed the operations. Li S: collected the data. Jiang C: conceived the study. All authors read and approved the final manuscript.
